# Whimbrel populations differ in trans-atlantic pathways and cyclone encounters

**DOI:** 10.1038/s41598-021-92429-z

**Published:** 2021-06-21

**Authors:** Bryan D. Watts, Fletcher M. Smith, Chance Hines, Laura Duval, Diana J. Hamilton, Tim Keyes, Julie Paquet, Lisa Pirie-Dominix, Jennie Rausch, Barry Truitt, Brad Winn, Paul Woodard

**Affiliations:** 1 Center for Conservation Biology, College of William & Mary, Williamsburg, VA USA; 2 Mount Allison University, Sackville, NB Canada; 3 Georgia Department of Natural Resources, Wildlife Resources Division, Non-Game Conservation Section, Brunswick, GA USA; 4 Canadian Wildlife Service, Environment and Climate Change Canada, Sackville, NB Canada; 5 Canadian Wildlife Service, Environment and Climate Change Canada, Iqaluit, NT Canada; 6 Canadian Wildlife Service, Environment and Climate Change Canada, Yellowknife, NT Canada; 7 The Nature Conservancy’s Virginia Coast Reserve, Nassawadox, VA USA; 8 Manoment Inc., Manomet, MA USA

**Keywords:** Ecology, Climate sciences

## Abstract

Each year hundreds of millions of birds cross the Atlantic Ocean during the peak of tropical cyclone activity. The extent and consequences of migrant-storm interactions remain unknown. We tracked whimbrels from two populations (Mackenzie Delta; Hudson Bay) to examine overlap between migration routes and storm activity and both the frequency and consequence of storm encounters. Here we show that Mackenzie Delta and Hudson Bay whimbrels follow different routes across the ocean and experience dramatically different rates of storm encounters. Mackenzie Delta whimbrels departed North America from Atlantic Canada, made long ($\bar{x}$ = 5440 ± 120.3 km) nonstop flights far out to sea that took several days ($\bar{x}$ = 6.1 ± 0.18) to complete and encountered storms during 3 of 22 crossings. Hudson Bay whimbrels departed North America from the south Atlantic Coast, made shorter ($\bar{x}$ = 3643 ± 196.2 km) nonstop flights across the Caribbean Basin that took less time ($\bar{x}$ = 4.5 ± 0.29) to complete and encountered storms during 13 of 18 crossings. More than half of Hudson Bay storm encounters resulted in groundings on Caribbean islands. Grounded birds required longer ($\bar{x}$ = 30.4 ± 5.32 days) to complete trans-Atlantic crossings and three were lost including 2 to hunters and 1 to a predator. One of the Mackenzie Delta whimbrels was lost at sea while crossing the Intertropical Convergence Zone. Whimbrels use two contrasting strategies to cross the Atlantic including (1) a long nonstop flight around the core of storm activity with a low likelihood of encountering storms but no safety net and (2) a shorter flight through the heart of Hurricane Alley with a high likelihood of encountering storms and a safety network of islands to use in the event of an encounter. Demographic consequences of storm encounters will likely play a role in the ongoing evolution of trans-Atlantic migration pathways as global temperatures continue to rise.

A large number of bird species that breed in North America cross the Atlantic Ocean during the fall to reach winter areas in South America^1^. For many species, transoceanic flights coincide with the late summer peak in tropical cyclone activity within the Atlantic Basin^2^. Annually, a large portion of Atlantic cyclones form as disturbances along the west coast of Africa, move west along the Intertropical Convergence Zone (ITCZ) and build in strength as they reach the western Caribbean^3^. This recurring distribution of storms suggests that birds flying over some waters would have a high likelihood of encountering storms while those flying over other waters would be able to avoid the largest concentration of high-intensity storms. To date, the relationship between migration pathways and storm exposure has not been explored and the frequency of storm encounters on the open ocean is wholly unknown. With projected increases in the intensity and frequency of strong Atlantic storms as global temperatures continue to rise^4, 5^, the demographic consequences of storm encounters may have implications for the ongoing evolution of migration routes and/or the persistence of migrant populations.

Whimbrels (*Numenius phaeopus hudsonicus*) in North America occur within three isolated breeding populations including within the lowlands of western Hudson Bay, throughout the Mackenzie River Delta in western Canada and in Alaska^6^. The Alaskan population migrates south along the Pacific Flyway to winter areas from Panama through Chile^6–8^. Both the Hudson Bay and Mackenzie Delta populations migrate south along the Western Atlantic Flyway to a common winter ground along the northern coast of South America^6, 9, 10^. Both of these populations make trans-Atlantic flights to reach winter grounds. Whether these populations use the same or different trans-Atlantic pathways, fly through the core of storm activity or experience similar storm encounter frequencies during migration remains unknown.

The relatively recent development of small tracking devices has opened the possibility of tracking small birds over large distances including the open ocean^11, 12^. The deployment of tracking devices on adult whimbrels from both the Mackenzie Delta and Hudson Bay populations and the accumulation of trans-Atlantic tracks over a period of twelve years now allows for an assessment of trans-Atlantic pathways and frequencies of storm encounters. We use migration tracks to compare (1) autumnal migration pathways, (2) the period of exposure to areas with the highest cyclone activity, (3) the rate of storm encounters and (4) the consequence of storm encounters between the Mackenzie Delta and Hudson Bay populations.

## Results

**Figure 1:**
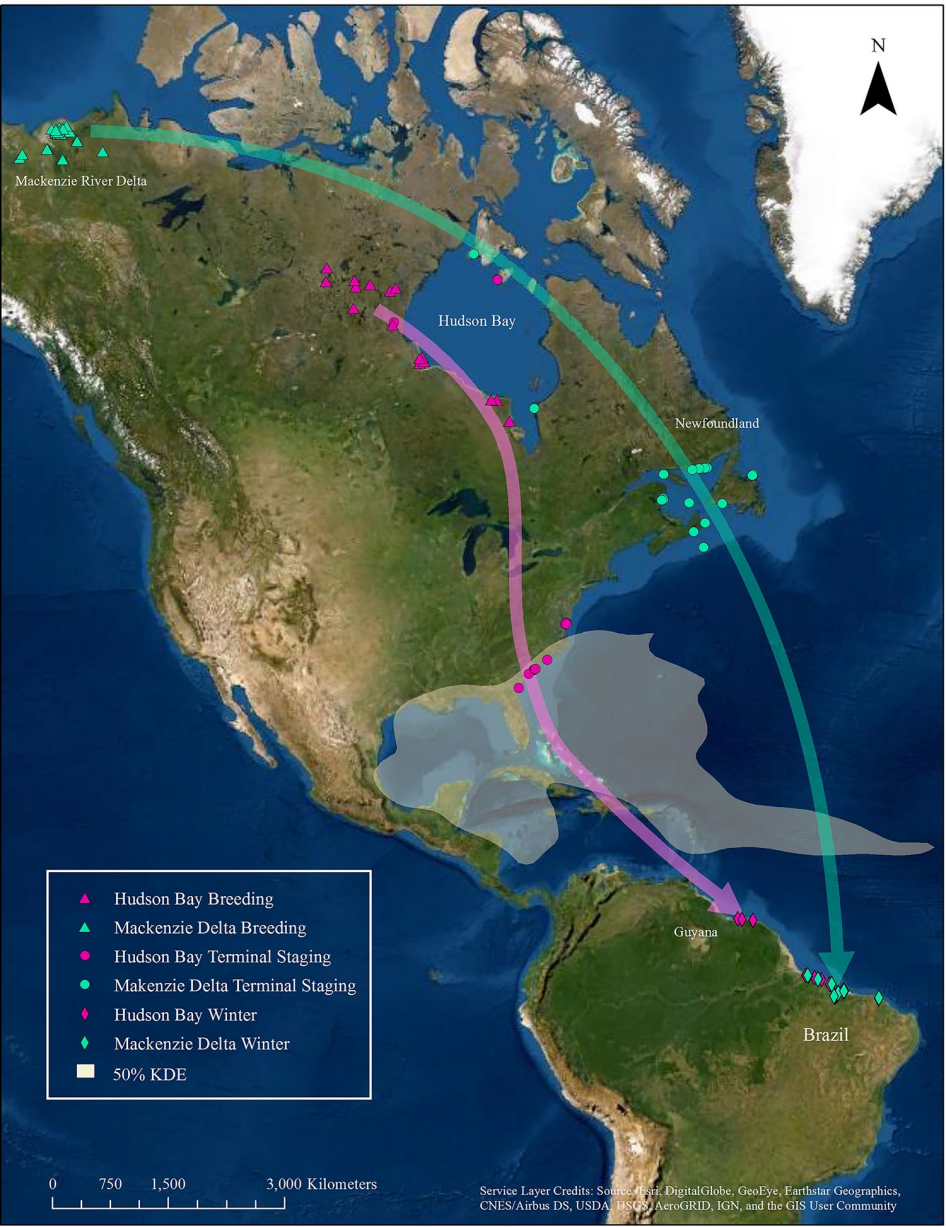
Stylized map of fall migration pathways for Mackenzie Delta and Hudson Bay populations and their overlap with the core area for tropical cyclone activity. Each triangle, circle, and diamond represent centroids for breeding territories, terminal staging locations, and wintering territories for each year and each individual. This figure was produced in ArcGIS 10.7.1 by an author: https://www.esri.com.

Mackenzie Delta and Hudson Bay populations followed different migration schedules and pathways between breeding and winter areas. Mackenzie Delta birds left their breeding grounds 18 days earlier (7 July ± 2.8 days; mean ± SE, n = 15) compared to Hudson Bay birds (25 July ± 3.5 days, n = 16) and arrived on winter territories 24 days earlier (7 September ± 4.4 days, n = 21 vs 2 October ± 7.5 days, n = 15). Following the breeding season, birds from Mackenzie Delta staged around the Beaufort Sea, flew east across the continent, staged in Atlantic Canada and then made nonstop, trans-oceanic flights to winter territories along the east coast of northern South America (Fig. 1). Following the breeding season, Hudson Bay birds staged around Hudson Bay and most flew southeast to stage along the south Atlantic Coast before flying across the Caribbean Sea to the northern coast of South America and then slowly east along the coast to winter territories. Some (12.8%) birds bypassed the south Atlantic Coast and flew directly across the Caribbean Sea. The total migration distance was longer (t-statistic = 24.9, df = 25, p < 0.001) for Mackenzie Delta birds ($\bar{x}$ = 11,012 ± 72.4 km, n = 14) compared to Hudson Bay birds ($\bar{x}$ = 8404 ± 76.0 km, n = 13). The longer distance and shorter duration resulted in a higher rate of advance toward winter grounds for Mackenzie Delta birds ($\bar{x}$ = 185 ± 11.8 km/day) compared to Hudson Bay birds ($\bar{x}$ = 155 ± 12.8 km/day).

### Trans-atlantic crossings

**Figure 2:**
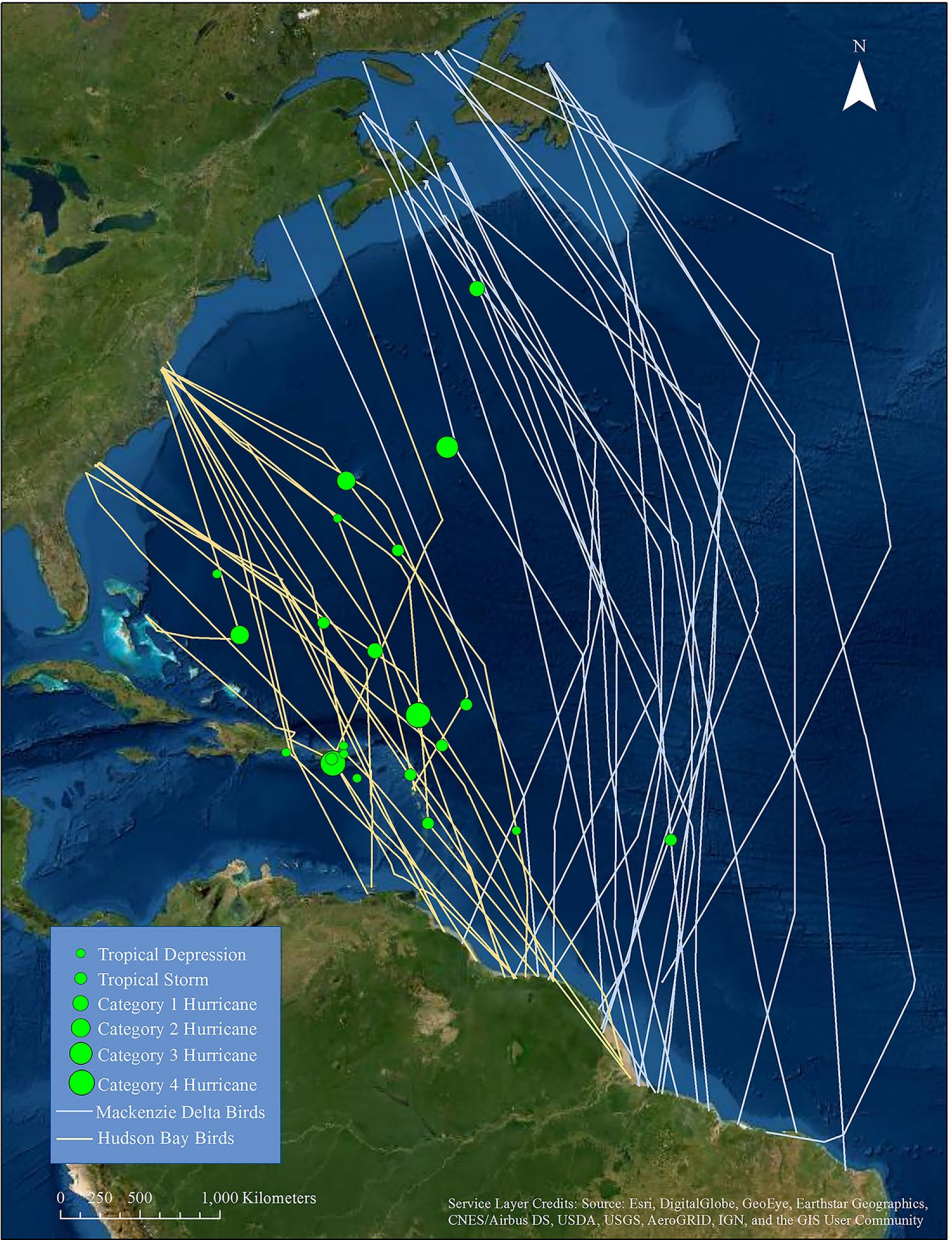
Tracks of autumn trans-Atlantic flights of whimbrels from Mackenzie Delta and Hudson Bay breeding populations. Tracks were recorded for birds fitted with solar-powered satellite transmitters. Symbols represent the locations of whimbrel encounters with tropical cyclones. This figure was produced in ArcGIS 10.7.1 by an author: https://www.esri.com/.

We recorded 47 transoceanic crossings (Fig. 2) involving 24 individual whimbrels (2008–2019). Birds from the Mackenzie Delta (n = 13) generally departed North America from the north Atlantic Coast ($\bar{x}$ = 47.5 ± 0.44 SE, latitude) and made long ($\bar{x}$ = 5440 ± 120.3, km) nonstop flights to South America that took several days ($\bar{x}$ = 6.1 ± 0.18, days) to complete. Birds from Hudson Bay (n = 11) generally departed from the south Atlantic Coast of North America ($\bar{x}$ = 37.0 ± 0.65, latitude) and made shorter ($\bar{x}$ = 3643 ± 196.2 km) nonstop flights to South America that took less time ($\bar{x}$ = 4.5 ± 0.29 days) to complete.

### Tropical cyclone exposure

Due to differences in trans-Atlantic routes, whimbrels from Hudson Bay had longer exposure to the core cyclone area (50% KDE) within the Atlantic Basin compared to whimbrels from Mackenzie Delta (Fig. 1). Hudson Bay birds that flew nonstop through the high-risk core area (polygon of storm kernel in Fig. 1) were exposed for 2.4 ± 0.18 days compared to only 0.4 ± 0.02 for birds from Mackenzie Delta (t-statistic = 14.3, df = 33, p < 0.001). Because most of the West Indies lie within the core cyclone area, birds that were grounded within the island group experienced very high exposure to cyclones. Time spent within the high-risk core area for grounded birds was 29.3 ± 7.78 days compared to only 2.4 ± 0.18 days for nonstop migrants (t-statistic = 5.8, df = 16, p < 0.001). The two populations also differed in where they crossed the core cyclone area. Hudson Bay birds crossed through the eastern region where they may encounter more powerful storms. Mackenzie Delta birds crossed through the Intertropical Convergence Zone where Atlantic cyclones are just forming as depressions or tropical storms.

### Tropical cyclone encounters

During 17 (36%) of the transoceanic crossings whimbrels encountered 22 storms including 16 while in flight and 6 after grounding on Caribbean islands. The likelihood of encountering a storm was influenced by population (mixed-effect logistic regression, z-statistic = − 3.15, p < 0.001) within our top model, which included population as a fixed effect and year as a random intercept. Only 3 (12%) of 26 transoceanic crossings by Mackenzie Delta birds encountered storms compared to 13 (62%) of 21 birds from Hudson Bay (g-statistic = 18.3, df = 1, p < 0.001). Storms encountered ranged in strength from tropical depressions to category 4 hurricanes (Fig. 2). Eight (73%) of 11 birds exhibited a detectable response to storms of tropical storm strength or higher while 2 (40%) of 5 birds responded to tropical depressions. Six birds exhibited no detectable response. Responses of flying birds to storm encounters included 2 birds that changed directions and detoured around storms and 8 birds that were grounded on Caribbean islands.

Birds from the two breeding populations differed in their access to islands that might be used during emergencies while out to sea. Birds from Mackenzie Delta used a trans-Atlantic route that was far out to sea with no land available. Birds from Hudson Bay flew over the West Indies such that islands were available, but there were consequences associated with landing on islands. Ten birds from Hudson Bay stopped on islands, including 8 that were put down by storms and 2 that landed without encountering a storm. Three of the birds that stopped on islands died, including 2 that were shot by hunters on Guadeloupe and 1 that was apparently taken by a raptor on Puerto Rico. Compared to birds that made nonstop flights ($\bar{x}$ = 4.5 ± 0.29 days, n = 11) over the Caribbean, grounded birds required significantly (t-statistic = − 7.01, df = 19, p < 0.001) longer periods ($\bar{x}$ = 30.4 ± 5.32 days, n = 10) to complete their migrations to South America. This additional time within the Atlantic Basin made the birds vulnerable to additional storm encounters. Five (71%) of the 7 birds that were not lost on islands were hit by storms while grounded.

## Discussion

The two whimbrel populations used distinctly different pathways to cross the Atlantic Ocean. The two pathways had different downstream consequences. Mackenzie Delta whimbrels followed a longer route over the ocean that required a much longer sustained flight to complete. The existence of an evolutionarily stable nonstop eastern migration route across the Atlantic with a departure location in Atlantic Canada was predicted forty years ago by radar observations^13, 14^ and simulations^15^ that assumed tailwind assistance during departure and en route course corrections to take advantage of tailwind assistance from prevailing trade winds. The whimbrel tracks recorded here followed successful simulated routes. One of the clear advantages of this route is that birds avoid most storms by flying around the core of tropical cyclone activity. Only 3 of 22 bird tracks along this route encountered a storm. Two of these tracks encountered storms while flying east shortly after departing North America. Storms that move north and survive into the cooler waters of the North Atlantic are the exception but these may become more common as sea surface temperatures rise^4^. The third encounter occurred just north of South America within the Intertropical Convergence Zone. Crossing this disturbance zone may be the most dangerous segment of the eastern route as strong easterlies form during the migration season from the convergence of northeasterly and southeasterly trade winds. These cross winds represent an energetically costly barrier to overcome^16^. Birds confront this barrier only after they have already flown nonstop for more than 4500 km. The only bird that died within this route was lost at sea while crossing the ITCZ. Birds using this route are far out to sea and have no safety net or place to land in the event of a storm encounter.

Hudson Bay whimbrels followed a shorter, more direct route across the ocean that included more storm hazards. Birds following this route that do not encounter storms make quick passage to South America. However, Hudson Bay birds fly the gauntlet through the heart of hurricane alley where storm encounters may be costly. More than half of all Hudson Bay tracks encountered storms. The route passes over the West Indies and islands provide a safety net in the event of a storm encounter. More than half of encounters resulted in birds grounding on islands. Groundings delay birds and extend the ocean crossing by a considerable amount, making them vulnerable to additional storm encounters. Although islands may provide a place to weather storms, they are not entirely safe. Some islands within the Lesser Antilles continue to support legal shorebird hunting^17^. Hurricane-aided hunting represents a population-level threat to some shorebirds of conservation concern^18^. Two of the 8 birds grounded on the islands were shot by hunters in shooting swamps. Raptors also threaten grounded shorebirds during migration^19, 20^. The transmitter of a third bird lost within this route was recovered from the roof of a building in San Juan, Puerto Rico; this bird was believed to have been taken by a raptor while grounded.

**Figure 3:**
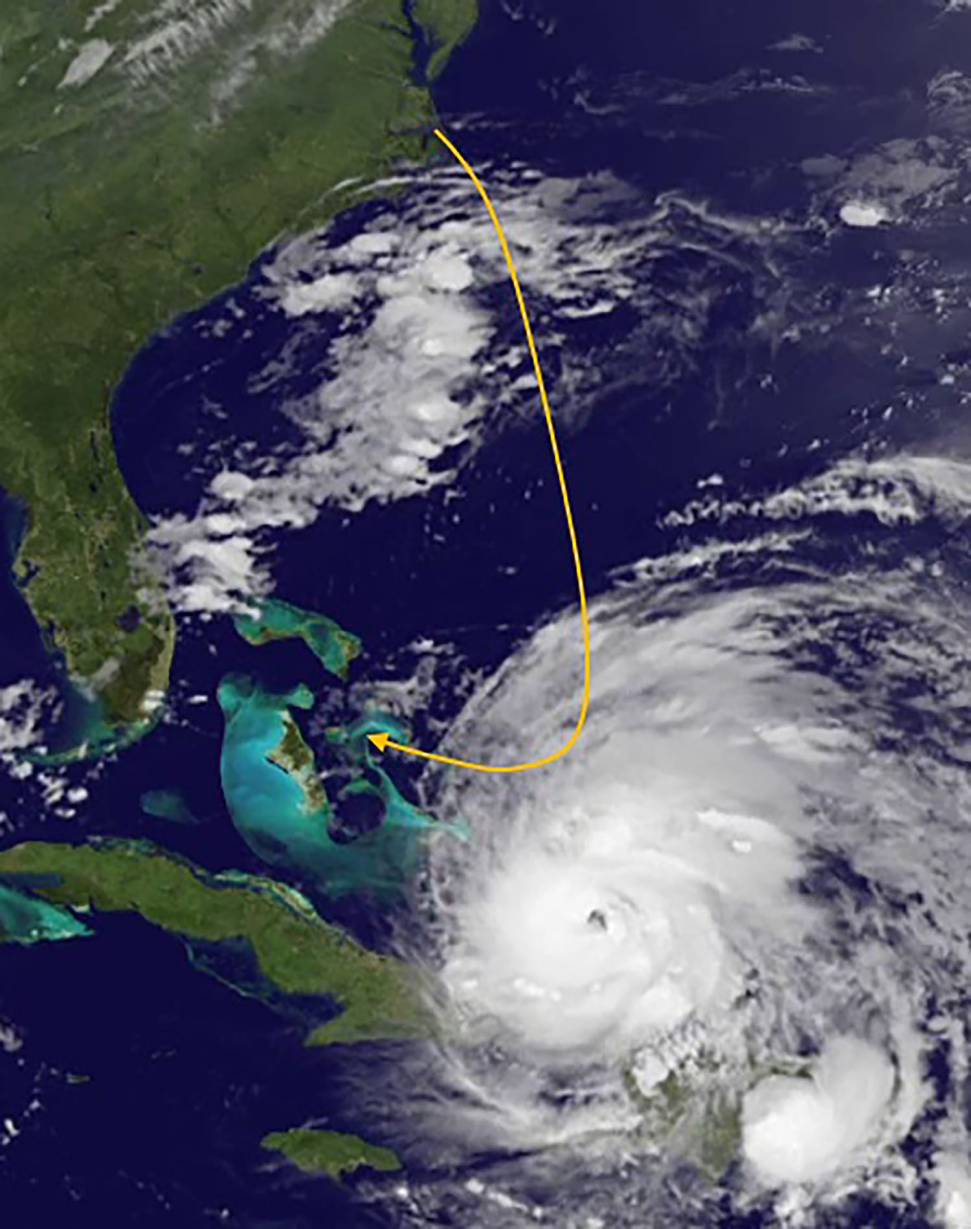
Stylized encounter between a Hudson Bay whimbrel and Hurricane Irene on 24 August 2011. Irene was a category 2 hurricane when encountered. The bird would retreat more than 400 km and put down on North Eleuthera in the Bahamas. The bird staged on the Bahamas for 19 days before flying to Puerto Rico where it was hit by Tropical Storm Maria. The bird staged on Puerto Rico for 17 days before leaving and completing migration to South America. This figure was created from imagery gathered from non-copyrighted NOAA Satellite Maps application for the now retired GOES-13 satellite: https://www.star.nesdis.noaa.gov/GOES/index.php.

Whimbrels appear capable of enduring some storms en route on the open ocean, though there is a limit to what they can manage. Four birds flew directly through tropical depressions or tropical storms and two birds flew through the outer bands of category 1 and 3 hurricanes and continued on to complete migration. Although these birds did not exhibit immediate responses to the interaction, some of these birds turned from their initial heading later in migration to make an earlier landfall (Fig. 2). These late detours suggest that storm encounters likely impacted their energy budgets, forcing them to make route adjustments. Conversely, the large hurricanes that build within the western Caribbean represent un-navigable hazards to whimbrels migrating through this region (Fig. 3). It is unlikely that birds can fly through these wind fields and many of these storms are large enough that detouring around them is not possible. It seems likely that the availability of islands offering refugia from these storms may contribute to the viability of the western route. With the exception of birds that were lost on islands, all birds that encountered storms and were grounded continued on to complete migration.

The demographic consequences of storm encounters will likely play a role in the ongoing evolution of trans-Atlantic migration pathways. Storm dynamics are complex^21^. Both the spatial and temporal patterns of storms are subject to several forcing factors including global warming^5^. Storm intensity is partially the product of sea-surface temperature which is expected to continue rising with global warming^22^. Increases in storm size and intensity may result in more Hudson Bay whimbrels being grounded within the Caribbean Basin. With sea-surface temperatures increasing throughout higher latitudes, the number of storms that maintain strength and extend into the North Atlantic is also expected to rise^22, 23^. Strenuous encounters with storms in the North Atlantic near the beginning of a 5000-km nonstop flight may impair the ability of Mackenzie Delta whimbrels to reach South America. Frequent encounters within this area may effectively close off the eastern route during periods of high storm activity. Changes in storms over time may force whimbrels and other migratory species to make adjustments in migration pathways and/or phenology. Whimbrel populations using the Western Atlantic Flyway are experiencing a high rate of decline^24^ and have been classified as populations of High Conservation Concern^25^.

## Methods

### Field methods

We captured 24 whimbrels between 2008 and 2018. Birds were captured on migration staging sites along the lower Delmarva Peninsula in Virginia, USA (*n* = 6) (37.398° N, 75.865° W), along the coast of Georgia, USA (*n* = 5) (31.148° N, 81.379° W), along the Acadian Peninsula in New Brunswick, Canada (*n* = 3) (47.973° N, 64.509° W) as well as on the nesting ground near the Mackenzie River, Northwest Territories, Canada (*n* = 10) (69.372° N, 134.894° W). All birds were aged as adults by plumage^26, 27^ and were banded with United States Geological Survey tarsal bands and coded leg flags. Sex of captured birds was not determined.

We fitted all birds with satellite transmitters called Platform Transmitter Terminals (PTTs) using a modification of the leg-loop harness^28, 29^. Instead of elastic cord, we used Teflon^®^ ribbon (Bally Ribbon Mills, Bally, Pennsylvania, USA) that was fastened with brass rivets or crimps^30^. We glued transmitters to a larger square of neoprene to elevate it above the body and prevent the bird from preening feathers over the solar panels. The transmitter package was below 3% of body mass (measured at the time of deployment,$\bar{x}$ = 484.5 ± 17.1) for all individuals tracked in this study. The PTTs used in this study were 9.5 g PTT-100 (*n* = 14) or 5.0 g PTT-100 (*n* = 10) solar-powered units produced by Microwave Telemetry, Inc. (Columbia, Maryland, USA).

### Tracking

Birds were located using satellites of the National Oceanic and Atmospheric Administration and the European Organization for the Exploitation of Meteorological Satellites with onboard tracking equipment operated by Collecte Localisation Satellites (CLS America, Inc., Largo, Maryland, USA)^31^. Transmitters were programmed to operate with a duty cycle of 24 h off and 5 h on (*n* = 9) or 48 h off and 10 h on (*n* = 15) and collected 1–34 ($\bar{x}$ = 5.48 ± 0.07) locations per cycle. Locations in latitude and longitude decimal degrees, date, time, and location error were received from CLS America within 24 h of satellite contact with PTTs. Locations were estimated by the Advanced Research and Global Observation Satellite (ARGOS) system (www.Argos-system.org), which uses a Doppler shift in signal frequency and calculates a probability distribution within which the estimate lies. The standard deviation of this distribution gives an estimate of the location accuracy and assigns it to a “location class” (LC): LC3 =  < 150 m, LC2 = 150–350 m, LC1 = 350–1000 m, LC0 > 1000 m, LCA = location based on 3 messages and has no accuracy estimate, LCB = location based on 2 messages and has no accuracy estimate, and LCZ = location process failed. We used LC classes 1–3 to determine whimbrel locations.

### Migration pathways

We used tracking data to delineate fall migration pathways and, though migration duration can include fueling at breeding territories^32^, we defined migration duration as the time between departure from the breeding grounds and arrival on winter territory. We identified the source population for all individuals included in this study either by capture on the breeding grounds (*n* = 10) or by capture within migratory staging sites and tracking birds to the breeding grounds (*n* = 14). Birds were either from the Mackenzie Delta (*n* = 13) or Hudson Bay (*n* = 11) breeding populations. We assessed departure and arrival when birds moved away from or settled into stationary breeding and winter territories respectively. Departure was abrupt and we recorded no “false starts” of birds leaving breeding areas and then returning before resuming migration. We present a stylized map of migration routes that was drawn by hand using the collection of flights recorded to provide a broad overview of routes relative to the distribution of storms.

### Trans-atlantic flights

We used tracking data to delineate migration pathways across the Atlantic Ocean (from coast of North America to coast of South America). Most birds departed from coastal staging sites and we considered the last staging location prior to crossing the Atlantic the terminal staging area. Several birds departed from inland locations on James Bay. We only consider the segment of the latter flights that occur over the ocean. We consider the duration of transoceanic flights to be the time interval between emerging from the coast of North America and arriving along the coast of South America. In cases where departure and arrival times occurred outside the radio transmitter’s duty cycle, we drew a straight-line between the last known location on land for departures or the first known location on land for arrivals and the nearest location over water and measured the distance between the in-flight point and the coastline along the line. We then used the mean overall speed between in-flight points for all birds ($\bar{x}$ = 14.8 ± 0.4 m/s, n = 40) to interpolate the leaving or arrival times. We consider the flight length to be the sum of the distance between consecutive locations along the path taken between the site of emergence along the coast of North America and the site of landfall along the northern coast of South America.

### Exposure to tropical cyclones

We examined the distribution of tropical cyclones throughout the Atlantic Ocean using position records (1961–2018) within the revised Atlantic hurricane best tracks from the National Hurricane Center (https://www.nhc.noaa.gov/data/#hurdat), known as the Atlantic HURDAT2^33^. We restricted our analyses to storms classified as tropical depressions or above and HURDAT data collected since 1961, when satellites were first used to monitor tropical cyclone activity^34^. The database contains the storm category (Saffir Simpson Scale), wind speed (mph) and coordinates recorded for six-hour intervals during the period that each storm existed using standard six-hour intervals which allows for weighting of the storms according to their lifespans and estimating the distribution of probability density. We selected storms (*N* = 590) that were active between 15 July and 30 November to coincide with whimbrel migration through the region. We mapped all storm observation points (*N* = 17,637) using a kernel density estimator (KDE) method^35^ with the “ks” package^36^ in program R^37^. We used the normal (or Gaussian) kernel and a smooth cross-validation bandwidth selector^38^ to map 50% kernel densities. We considered the 50% KDE to be the area of highest storm occurrence and estimated exposure to this region by overlaying whimbrel tracks on the KDE polygon and measuring each whimbrel’s time within the area. Because the first and last points within the polygon occurred when the bird’s transmitter first transmitted the bird’s location within and outside the polygon, rather than when the bird first entered and exited the polygon, we measured the distance between the first point inside the polygon and the previous point outside the polygon and used the mean overall speed between in-flight points for all birds (,$\bar{x}$ = 14.8 ± 0.4 m/s, n = 40) to interpolate the time that the bird entered the polygon. We used the same method to calculate the time that the bird left the polygon using the last point within the polygon and next point outside the polygon.

### Encounters with tropical cyclones

We documented encounters between whimbrels and tropical cyclones within the Atlantic Basin by overlaying migration tracks for individual birds on archives of storm tracks within HURDAT2 for the period (2008–2019) of the tracking study. We considered a whimbrel-storm encounter to have occurred when bird tracks intersected storm tracks during the same time period. For grounded birds, we considered an encounter to have occurred when a storm track moved over the ground position of a bird. For each encounter, we recorded the coordinate of the encounter and the storm intensity. Storm intensities were classified as tropical depressions, (≤ 38 mph), tropical storms (39–73 mph), category 1 hurricane (74–95 mph), category 2 hurricanes (96–110 mph), category 3 hurricanes (111–129 mph), category 4 hurricanes (130–156 mph), and category 5 hurricanes (≥ 157 mph) according to the Saffir–Simpson Hurricane Wind Scale^39^.

We examined the post-encounter track of birds to categorize the response of birds including none, detour or grounding. We considered birds to exhibit no response to the storm encounter if the migration trajectory was unchanged during or shortly following a storm encounter. We considered birds to have taken a detour in response to a storm encounter if the migration trajectory followed over the previous day was deflected by > 20° during or shortly following an encounter. We considered birds to have grounded if they landed on an island following a storm encounter.

### Statistics

We used mixed-effects logistic regressions (R3.6.2: R Core Team 2019) to compare the likelihood of storm encounters between whimbrel populations using tracks as replicate samples. We initially fit models using whimbrel identity and year as random intercepts to account for potential lack of independence for journeys made by the same individuals and journeys made within the same year, but inclusion of bird identity as a random intercept resulted in a singular fit so this variable was excluded from further analysis. We then compared models with year as a random intercept and no fixed effects, year as a random intercept and breeding population (Mackenzie Delta vs Hudson Bay) as a fixed effect, year as a random intercept and journey number (1st, 2nd, or 3rd journey) as a fixed effect, and year as a random intercept with breeding population and journey number as fixed effects. We used Akaike’s information criterion for small sample size (AIC_c_) and selected the model with the lowest AIC_c_ score as the best-supported model if no other model was within 2 ΔAIC_c_ after removing models with uninformative parameters^40^. Several birds made more than one transoceanic crossing in different years and we consider these to be independent samples. We used two-tailed t-tests to compare migration lengths and duration between routes. We used g-tests with Yates correction to make frequency comparisons.

### Data and ethics statement

This study was conducted in compliance with ARRIVE guidelines. Data used in this manuscript are unique and have not been submitted for publication elsewhere. The authors claim no conflict of interest. This project was reviewed and approved by the William & Mary Institutional Animal Care and Use Committee protocol IACUC-2017-04-18-12065 of The College of William and Mary, Environment Canada Animal Care Committee protocols EC-PN-12-006, EC-PN-13-006, EC-PN-14-006, Mount Allison University Animal Care Committee protocol 15-14, and the Government of the Northwest Territories Wildlife Care Committee protocol NWTWCC2014-007. All Methods were performed in accordance with the relevant guidelines and regulations.

## Data Availability

Data used in this study reside within structured repositories. Whimbrel tracking data are archived within the online repository Movebank https://www.movebank.org/cms/movebank-main. Storm data are available through the online repository maintained by NOAA’s hurricane center https://www.nhc.noaa.gov/.

## Code availability

Data analyses were performed in Program R (v4.03). The code used for analyses is publicly available through the R packages 'ks' which was used to calculate KDE polygons, 'geosphere' which was used to calculate distances, and 'tidyverse' which was used to format and shape data. Arc GIS (v10.4) was used to measure time and distance within KDE polygons.
